# Chronic Insufficient Sleep Has a Limited Impact on Circadian Rhythmicity of Subjective Hunger and Awakening Fasted Metabolic Hormones

**DOI:** 10.3389/fendo.2018.00319

**Published:** 2018-06-12

**Authors:** Andrew W. McHill, Joseph T. Hull, Ciaran J. McMullan, Elizabeth B. Klerman

**Affiliations:** ^1^Division of Sleep and Circadian Disorders, Department of Medicine, Brigham and Women’s Hospital, Boston, MA, United States; ^2^Division of Sleep Medicine, Harvard Medical School, Boston, MA, United States; ^3^Oregon Institute of Occupational Health Sciences, Oregon Health & Science University, Portland, OR, United States; ^4^Renal Division, Brigham and Women’s Hospital, Boston, MA, United States; ^5^Channing Division of Network Medicine, Brigham and Women’s Hospital, Boston, MA, United States

**Keywords:** sleep restriction, appetite, sleep loss, endocrinology, forced desynchrony, circadian rhythms, fasting hormones, hunger

## Abstract

Weight gain and obesity have reached epidemic proportions in modern society. Insufficient sleep—which is also prevalent in modern society—and eating at inappropriate circadian times have been identified as risk factors for weight gain, yet the impact of chronic insufficient sleep on the circadian timing of subjective hunger and physiologic metabolic outcomes are not well understood. We investigated how chronic insufficient sleep impacts the circadian timing of subjective hunger and fasting metabolic hormones in a 32-day in-laboratory randomized single-blind control study, with healthy younger participants (range, 20–34 years) randomized to either Control (1:2 sleep:wake ratio, 6.67 h sleep:13.33 h wake, *n* = 7, equivalent to 8 h of sleep per 24 h) or chronic sleep restriction (CSR, 1:3.3 sleep:wake ratio, 4.67 h sleep:15.33 h wake, *n* = 8, equivalent to 5.6 h of sleep per 24 h) conditions. Participants lived on a “20 h day” designed to distribute all behaviors and food intake equally across all phases of the circadian cycle over every six consecutive 20 h protocol days. During each 20 h day, participants were provided a nutritionist-designed, isocaloric diet consisting of 45–50% carbohydrate, 30–35% fat, and 15–20% protein adjusted for sex, weight, and age. Subjective non-numeric ratings of hunger were recorded before and after meals and fasting blood samples were taken within 5 min of awakening. Subjective levels of hunger and fasting concentrations of leptin, ghrelin, insulin, glucose, adiponectin, and cortisol all demonstrated circadian patterns; there were no differences, however, between CSR and Control conditions in subjective hunger ratings or any fasting hormone concentrations. These findings suggest that chronic insufficient sleep may have a limited role in altering the robust circadian profile of subjective hunger and fasted metabolic hormones.

**Clinical Trial Registration:**

The study was registered as clinical trial #NCT01581125.

## Introduction

Over 30% of Americans are obese (1) and 1.4 billion adults worldwide are overweight (2, 3). High body weight is associated with an increased risk for heart disease, stroke, osteoarthritis, diabetes, and cancer (4) and accounts for ~147 billion dollars in health-care costs in the United States per year (5). Thus, identifying risk factors, behaviors, and mechanisms that promote weight gain is vital to help combat disease and associated decreases in quality of life for patients and their families.

Sleep is a vital component for the optimal functioning of many physiological processes such as metabolism (6), immune function (7), and cognition (8). A recent health survey reported that ~30% of Americans typically sleep less than 6 h per night (9), an amount far below what is needed for optimal health. Epidemiological evidence suggests that shorter sleeping durations are associated with higher body weight (10–12) and poorer glucose tolerance (13). Controlled in-laboratory studies have found that when sleep is acutely restricted and food is provided *ad libitum*, participants consume more nighttime calories than when they are attaining non-restricted sleep (14–17). It is not mechanistically clear, however, why sleep restriction increases caloric intake. Several studies have found that acute sleep restriction can lead to changes in appetitive hormones that would stimulate hunger: specifically, a decrease in the satiety hormone leptin (10, 18–20) and an increase in the appetitive hormone ghrelin (10, 19, 21, 22). If food is provided *ad libitum*, however, participants during acute sleep restriction eat far more calories than needed to meet energy balance, despite increased leptin and decreased ghrelin concentrations (15, 16, 23), thus bringing the hormonal mechanism for increased food intake into question (24). It is not well understood how the levels of these hormones will react to chronic exposure to sleep restriction, which is a common cause of sleep loss and may be more important for metabolic health, as appreciable weight gain occurs chronically (25).

Circadian timing also plays a role in weight gain, subjective hunger, and diabetes risk. Individuals who work during the night, thereby shifting the timing of the majority of their caloric consumption to the nighttime hours when the internal biological clock is promoting sleep and fasting, have increased risk for obesity (26–28) and diabetes (29). Individuals who do not work during the night but eat closer to and during their biological nights (i.e., when the hormone melatonin is high) have higher percentages of body fat (30), potentially due to decreased metabolic rate during that time (31–33). The increased food consumption during the evening is driven by the circadian clock: in controlled in-laboratory settings, hunger ratings on a visual analog scale show robust circadian rhythmicity, peaking at a circadian phase equivalent to ~2000 hours and a trough at ~0750 hours (34). The appetitive hormones leptin and ghrelin have also been found to follow diurnal rhythms (35–38), with large influence from caloric intake (39) and sleep (36, 37, 40). Furthermore, under tightly controlled constant routine conditions when snacks are provided evenly every hour and constant wakefulness and posture are maintained (41), insulin concentrations follow a circadian rhythm, with a peak in the early biological morning (42, 43).

In a protocol combining sleep restriction with circadian disruption, Buxton and colleagues found that during sleep restriction and circadian disruption within a forced desynchrony (FD) protocol—a protocol designed to distribute all behaviors evenly across all phases of the circadian cycle—there were significant circadian rhythms in fasted glucose, insulin, and cortisol, with fasted insulin displaying lower levels after weeks of sleep restriction and no significant difference in fasted glucose, cortisol, or leptin concentrations (31). However, in that study, there was no control group that did not receive sleep restriction independent of circadian disruption, and thus it is difficult to conclude whether the changes in these hormones were actually due to the sleep restriction or the combination of sleep restriction and circadian disruption.

Whether there are hormonal changes during chronic sleep restriction (CSR) that may promote increased hunger and diabetes risk, and how the circadian timing of these hormones interact with CSR, remains unknown. Thus, the aim of the current study was to determine the impact of CSR and circadian timing on subjective hunger and fasted concentrations of hormones that may influence subjective hunger as compared to a Control condition. Furthermore, we wanted to examine the influence of CSR on fasted glucose and insulin concentrations along with the hormones adiponectin and cortisol, which influence glucose tolerance.

## Materials and Methods

### Participants

Seventeen healthy participants completed a 32-day inpatient protocol, but due to blood sample collection issues in 2 participants (one in the Control and one in the CSR condition), data from 15 participants (7 males) are presented in the current study [BMI, 23.6 ± 3.7 kg/m^2^, 18.2–28.4 kg/m^2^; weight, 64.9 ± 12.0 kg, 47.2–88.9 kg; age 26.7 ± 4.4 years, 20.0–34.0 years; (mean ± SD, range)]. Participants were deemed medically and psychologically healthy based on self-reported health, psychological screening questionnaires, physical examination by a physician, laboratory testing of hematological or metabolic measures, and psychological evaluation from a clinical interview with a psychologist. Participants were also free of any sleep disorders, as determined by questionnaires and an overnight clinical sleep screening. Exclusion criteria consisted of a self-reported habitual sleep duration <7 or >9 h averaged across the entire week, history of night-shift work or transmeridian travel <3 months prior to study, BMI <18 or >29.9, age <18 or >35 years, pregnancy, and use of any prescription medication. For at least 3 weeks prior to the inpatient protocol, participants maintained an approximate 10-h per night sleep schedule at their self-reported habitual timing that was verified by sleep logs, wrist actigraphy (Actiwatch-L Mini Mitter/Respironics), and call-ins to a time-stamped voicemail recording system immediately upon awakening and prior to going to bed. This ~3-week sleep-wake schedule was implemented to ensure participants were not sleep restricted prior to admission to the Brigham and Women’s Hospital Center for Clinical Investigation research facility. In addition, participants abstained from any over-the-counter medication or drug use, alcohol, caffeine, nicotine, or other foreign substances during the ~3 weeks of at-home monitoring and for the duration of the protocol; this was verified *via* urine toxicology upon admission to laboratory. All participants provided written informed consent and all study procedures were approved by the Partners Healthcare Institutional Review Board. The study was registered as on clinicaltrials.gov as NCT01581125.

### Inpatient Protocol

**Figure 1:**
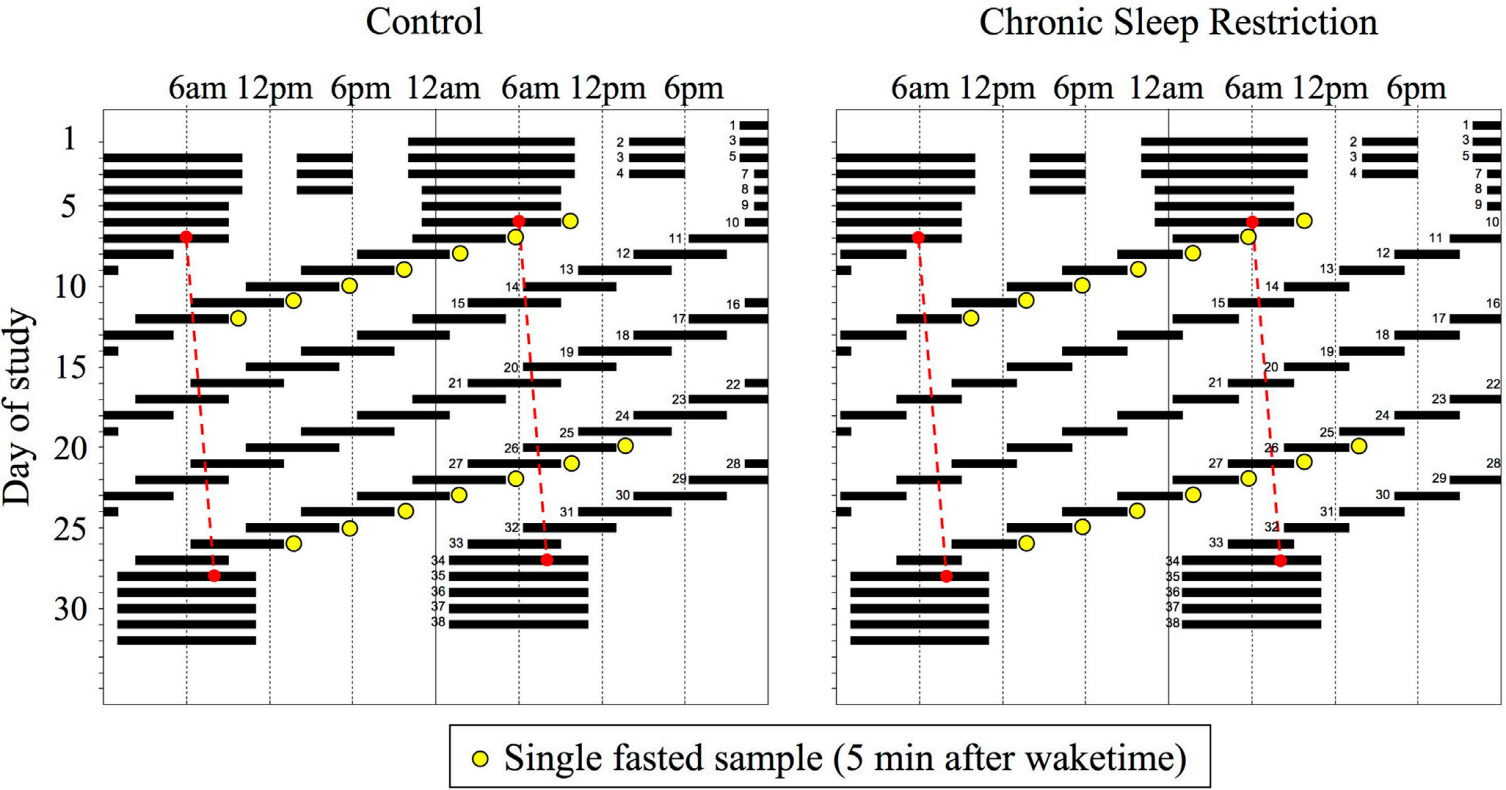
Raster plot of the Control (*n* = 7, 1:2 sleep:wake ratio, equivalent to 8 h sleep opportunity per 24 h day) and chronic sleep restriction (*n* = 8, 1:3.3 sleep:wake ratio, equivalent to 5.6 per 24 h day) forced desynchrony protocols. Clock hour is plotted on the horizontal axis and day of study on the vertical axis. Solid black bars represent sleep opportunities, yellow circles represent fasted blood draws, and the red dashed line represents the daily marker of a participant’s circadian period. Days are double plotted such that each consecutive study day is plotted next to and below the previous day.

Participants were admitted to a sound-attenuated, temperature-controlled suite that was free from time cues. All events were scheduled related to the participant’s habitual sleep-wake timing as determined from the ~3 weeks of at-home monitoring. Lighting was maintained at dim (<4 lux) levels during scheduled wakefulness and 0 lux during scheduled sleep. The first 3 days of the protocol were “sleep-satiation” days consisting of 12-h overnight sleep opportunities and 4-h daytime sleep opportunities to minimize any potential residual sleep loss upon entering the study. The next two “baseline” days consisted of 10-h overnight sleep opportunities at habitual timing. Participants were then scheduled to 24 cycles of a 20-h FD protocol and randomized to one of two sleep:wake FD conditions: Control (1:2, 6.67 h sleep opportunity, 13.33 h wake, equivalent to 8 h of sleep per 24 h day; seven participants) or CSR (1:3.3, 4.67 h sleep, 15.33 h wake, equivalent to 5.6 h of sleep per 24 h day; eight participants) (Figure 1). Use of the 20-h FD design allowed for the endogenous circadian pacemaker to run at each participant’s endogenous circadian period and uncouple the timing of each fasted blood draw from circadian phase. The participants were blinded to which condition they were randomized and the specifics of the FD protocol (e.g., time of day, day length, and date). Following the 24 cycles of FD, participants were provided five recovery days of 10 h sleep opportunities at the same circadian phase as the baseline sleep opportunities. During scheduled wakefulness, participants were allowed to engage in sedentary activities (e.g., read, talk or play board games with a researcher, and watch movies) and wakefulness was verified by continuous monitoring by research staff and continuous polysomnographic recordings.

During each 20 h day, participants were provided a nutritionist-designed, isocaloric diet consisting of 45–50% carbohydrate, 30–35% fat, and 15–20% protein adjusted for sex, weight, and age using the Harris–Benedict equation with an activity factor of 1.3 (44). During each wake episode, participants were provided a breakfast, lunch, and dinner and were instructed to consume all food provided. Subjective hunger was assessed using a visual analog scale that was given to each participant prior to and after each meal. The visual analog scale prompted the participant to identify on a 100 mm horizontal line how they felt at that moment, with each end of the line labeled with the extremes of the subjective continuum of “not at all hungry” to “extremely hungry.” Participants were not given any feedback on the “value” that they identified as using this scale.

Blood for fasted hormone concentrations was drawn *via* an indwelling venous catheter approximately 5 min after scheduled awakening (Figure 1). Prior to the blood draw, participants remained seated in bed, but were elevated to a semi-recumbent posture for cognitive computer testing. For the current analysis, data are from the first (study days 10–16) and last (study days 27–33) weeks of the FD protocol. Blood for melatonin concentrations was drawn hourly to enable assessment of circadian phase.

### Assay Information

All assays were performed by the Brigham and Women’s Hospital Research Assay Core; the Core was blinded to study condition. Leptin and active ghrelin were assayed using serum radioimmunoassay techniques (Millipore Research, St. Charles, MS, USA). The leptin assay had a sensitivity of 0.1 ng/mL, within assay coefficient of variation (CV) 5.2–5.7%, and between assay CV of 3.2–8.9%; ghrelin had a sensitivity of 7.8 pg/mL, within CV of 6.5–9.5%, and between CV of 9.6–16.2%. Insulin and cortisol were assayed using Access Chemiluminescent Immunoassay (Beckman Coulter, Fullerton, CA, USA) techniques with the insulin assay having a sensitivity of 0.03 μIU/mL, within assay CV of 2.0–4.2%, and between CV of 3.1–5.6%. The cortisol assay had a sensitivity of 0.4 μg/dL, a within CV of 4.4–6.7%, and a between CV of 6.4–7.9%. Finally, adiponectin was assayed using enzyme-linked immunosorbent assay techniques (ALPCO Diagnostics, Salem, NH, USA) with a sensitivity of 0.10 μg/mL, within assay CV of 5.0–5.4%, and between assay CV of ~6%.

### Statistical Analysis

To determine circadian phase for each individual, non-orthogonal spectral analysis of hourly serum melatonin was used to estimate intrinsic circadian period and subsequent circadian phase (45), with melatonin maximum set to 0 circadian degrees, which is ~0300 hours for individuals in entrained conditions. Subjective hunger and fasted hormone concentrations were analyzed as both raw values and normalized within each participant (*z*-scored) and binned into 60° (~4 h) circadian bins. One participant in the CSR condition had raw insulin concentrations much higher than other participants and was identified as an extreme (interquartile range × 3) outlier. Therefore, the data from this participant were omitted from the analysis of raw insulin concentrations. Subjective hunger and hormone concentrations were analyzed using mixed-effects models with circadian phases and condition as fixed factors and participant as a random factor to account for inter-participant differences. All statistical analyses were performed using SAS 9.4.

## Results

### Impact of Sleep Restriction and Circadian Timing on Subjective Hunger and Appetitive Hormones

**Figure 2:**
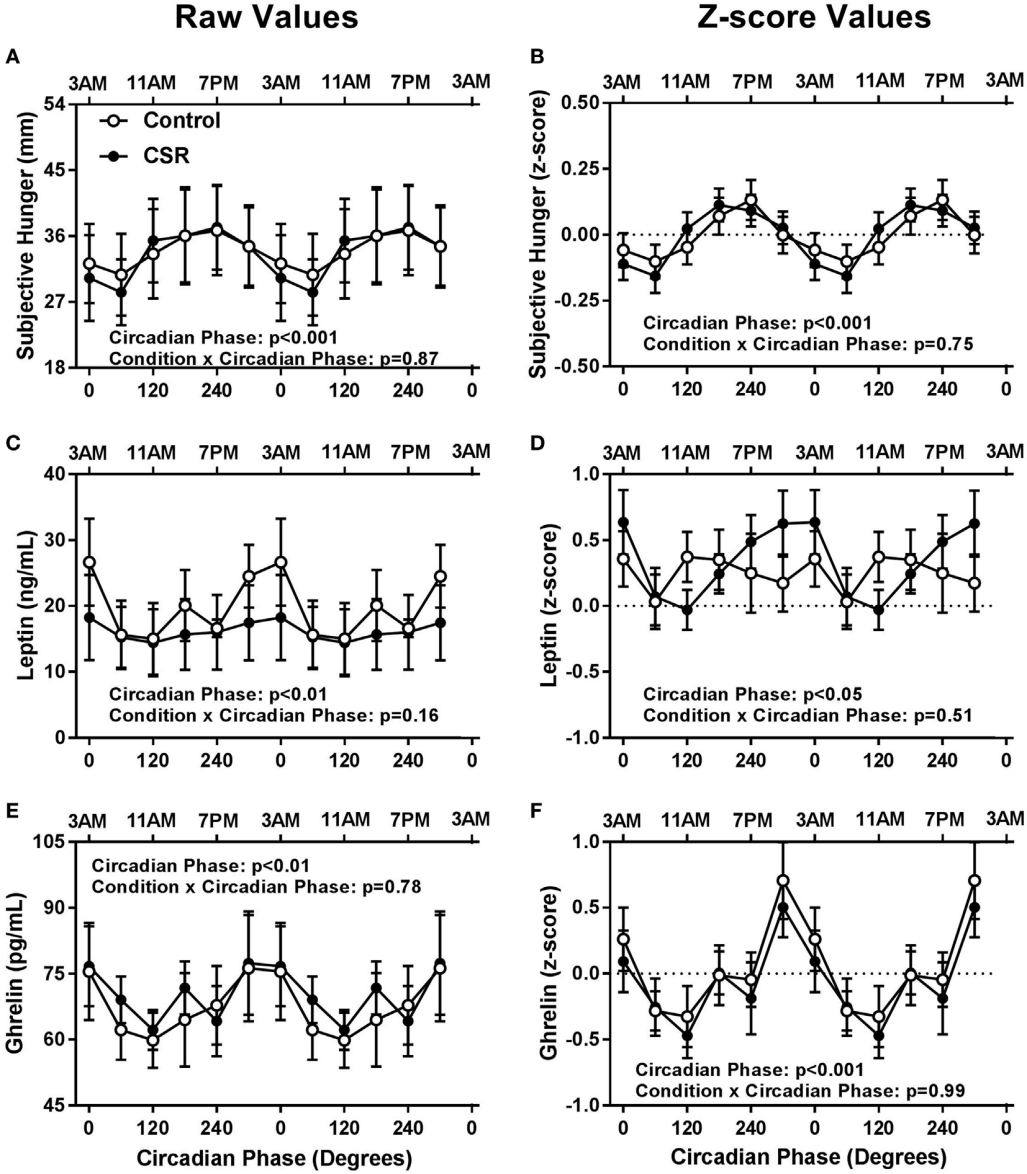
Influence of circadian phase and sleep restriction on **(A,B)** subjective hunger, **(C,D)** leptin, and **(E,F)** ghrelin. The Control (*n* = 7, 1:2 sleep:wake ratio, equivalent to 8 h sleep opportunity per 24 h day) condition is denoted by open circles and the chronic sleep restriction (CSR, *n* = 8, 1:3.3 sleep:wake ratio, equivalent to 5.6 h sleep opportunity per 24 h day) condition by closed circles. Data are represented as raw scores (left) and *z*-scores (right) and double plotted across circadian phase and relative clock hour for an individual with a habitual sleep time of 2300–0700 hours. Error bars represent SEM.

Contrary to expectations, there were no significant condition or condition-by-circadian phase interaction effects between CSR and Control conditions for subjective hunger, leptin, or ghrelin (Figure 2, all *p* > 0.16). There was, however, a robust circadian rhythm in subjective hunger (Figures 2A,B, both *p* < 0.001), such that subjective hunger levels peaked at 240° and reached a nadir at 60° for both Control and CSR conditions. Leptin, a satiety hormone, exhibited significant circadian rhythmicity (Figures 2C,D, both *p* < 0.05), with a peak at 0° and a nadir at 120° for both the Control and CSR conditions. Finally, ghrelin, an appetitive hormone, also displayed a significant circadian rhythm (Figures 2E,F, both *p* < 0.01) with a peak at 300° and nadir at 120° for both Control and CSR conditions. All findings were similar for raw and *z*-scored variables.

### Impact of Sleep Restriction and Circadian Timing on Insulin, Glucose, Adiponectin, and Cortisol

**Figure 3:**
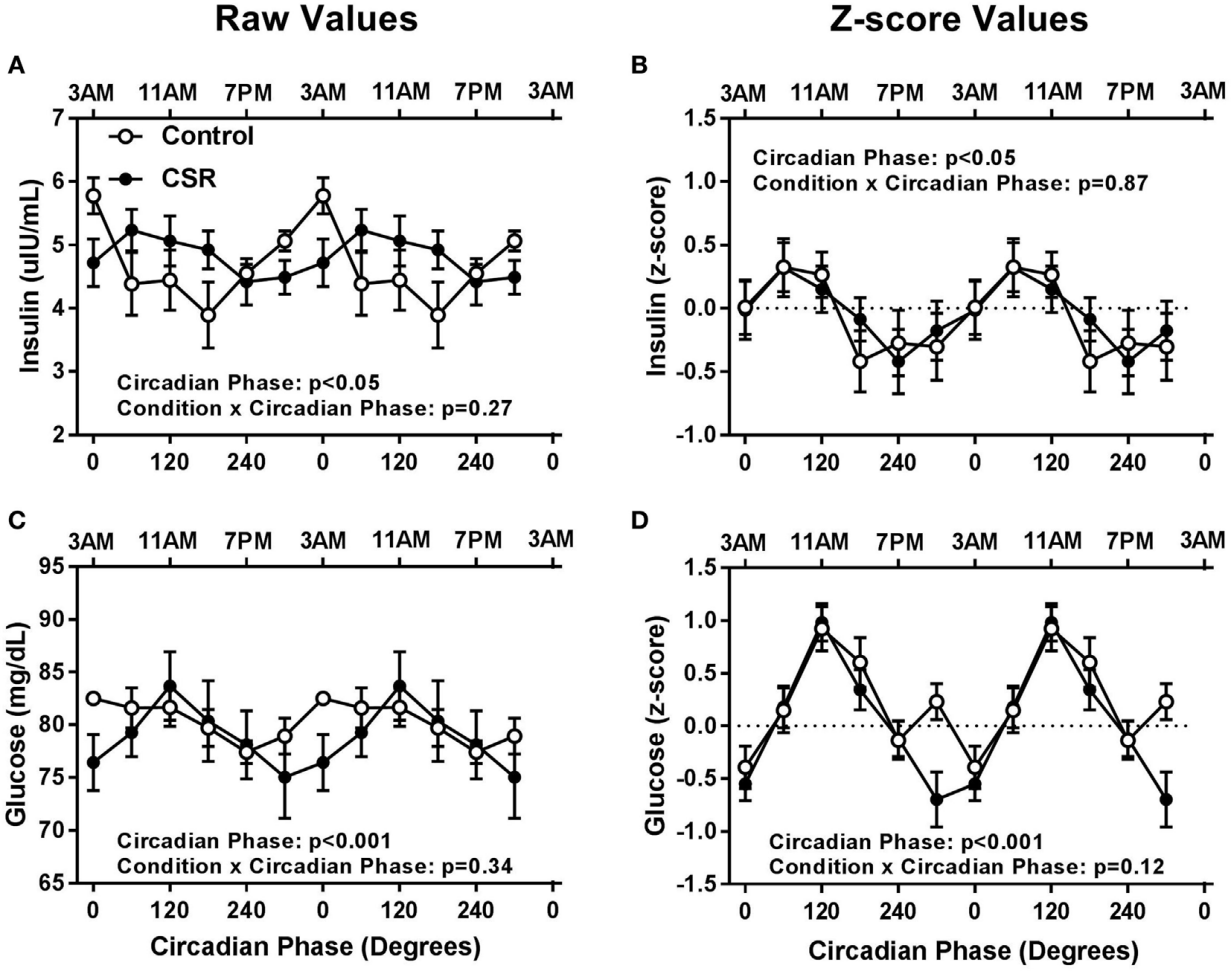
Influence of circadian phase and sleep restriction on fasted **(A,B)** insulin and **(C,D)** glucose concentrations. The Control (*n* = 7, 1:2 sleep:wake ratio, equivalent to 8 h sleep opportunity per 24 h day) condition is denoted by open circles and the chronic sleep restriction (CSR, *n* = 7, 1:3.3 sleep:wake ratio, equivalent to 5.6 h sleep opportunity per 24 h day) condition by closed circles. Data are represented as raw scores (left) and *z*-scores (right) and double plotted across circadian phase and relative clock hour for an individual with a habitual sleep time of 2300–0700 hours. Error bars represent SEM.

Somewhat surprisingly, fasted insulin and glucose levels did not have significant condition or condition-by-circadian phase interaction effects between CSR and Control conditions (Figure 3, all *p* > 0.12). Fasted insulin concentrations exhibited a significant circadian rhythm (Figures 3A,B, both *p* < 0.05) with a peak in concentrations between 0° and 60° and a nadir between 180° and 240° for both the Control and CSR conditions, while fasted glucose concentrations also displayed significant circadian variation (Figures 3C,D, both *p* < 0.001) with a peak at 0° for the Control condition and 120° for the CSR condition and a nadir between 240° and 300° for both the Control and CSR conditions. All findings were similar for raw and *z*-scored variables.

**Figure 4:**
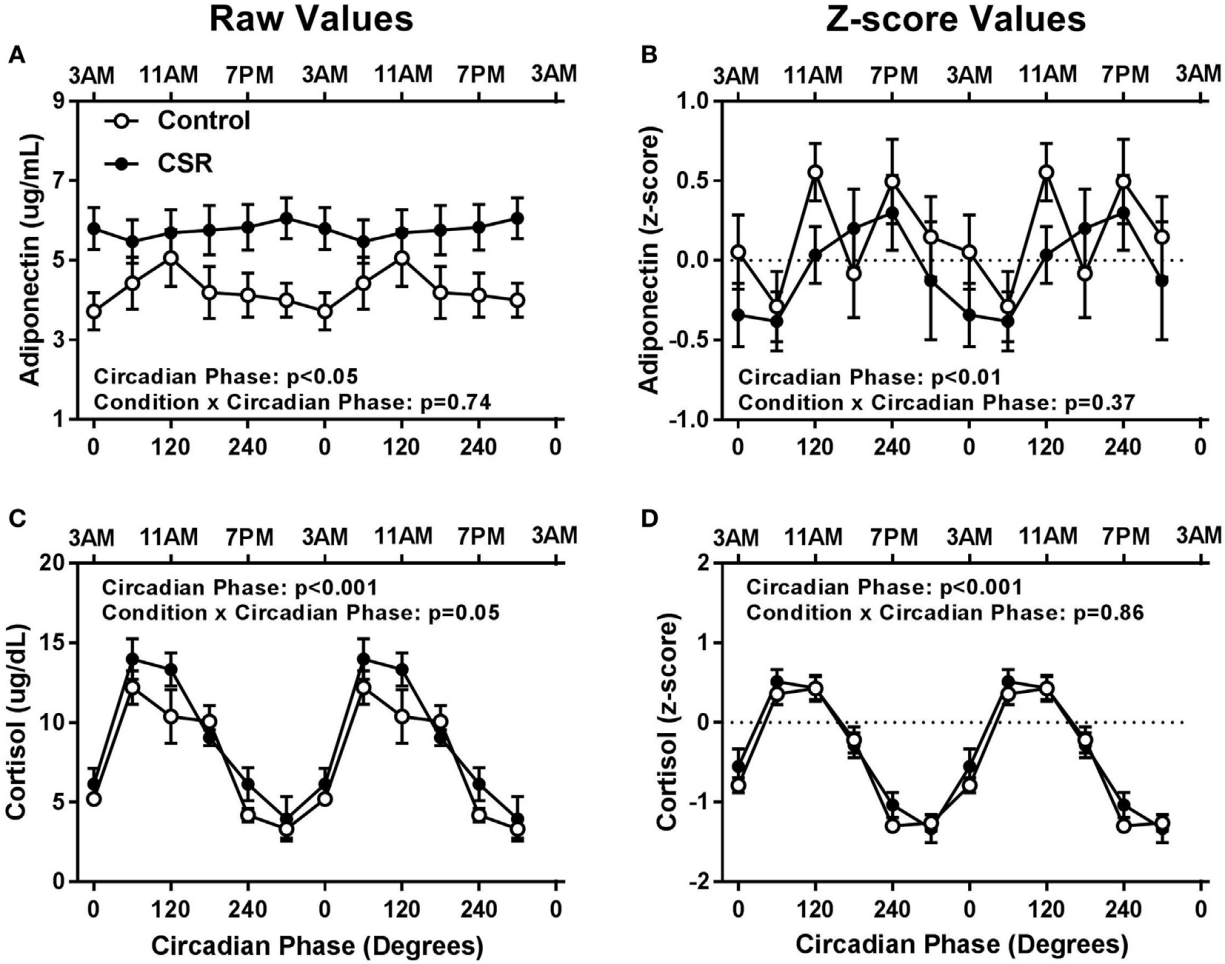
Influence of circadian phase and sleep restriction on fasted **(A,B)** adiponectin and **(C,D)** cortisol concentrations. The Control (*n* = 7, 1:2 sleep:wake ratio, equivalent to 8 h sleep opportunity per 24 h day) condition is denoted by open circles and the chronic sleep restriction (CSR, *n* = 8, 1:3.3 sleep:wake ratio, equivalent to 5.6 h sleep opportunity per 24 h day) condition by closed circles. Data are represented as raw scores (left) and *z*-scores (right) and double plotted across circadian phase and relative clock hour for an individual with a habitual sleep time of 2300–0700 hours. Error bars represent SEM.

Adiponectin concentrations did not have significant condition or condition-by-circadian phase interaction effects between CSR and Control conditions (Figures 4A,B, both *p* > 0.37). Cortisol concentrations also did not display significant condition effects (Figures 4C,D, both *p* > 0.18), but did display a non-significant trend for condition-by-circadian phase interaction effects for higher raw cortisol concentrations in the CSR condition (Figure 4C, *p* = 0.05). Fasted adiponectin concentrations did display a significant circadian rhythm (Figures 4A,B, both *p* < 0.05) with a peak at 120° and 240°, for the Control and CSR conditions, respectively and a nadir at 0°–60° for both conditions. Finally, fasted cortisol also exhibited an expected robust circadian rhythm (Figures 4C,D, both *p* < 0.001) with a peak in the early morning hours at 60° and a nadir during the evening at 300° for both conditions. All findings were similar for raw and *z*-scored variables.

## Discussion

Sleep restriction, experienced by millions of individuals on a daily basis, has been associated with weight gain and poor glucose tolerance. In-laboratory studies of *acute* sleep restriction have shown that weight gain occurs due to consumption of excess nighttime calories (14–17); however, the physiological drive for the excess of consumption of calories, particularly in response to chronic insufficient sleep, is unclear. In the current study, we show that there is limited evidence for a role of CSR in levels of subjective hunger, fasted appetitive hormones, and measures of fasted glucose metabolism, however, there is strong circadian phase modulation influencing these outcomes. These findings provide valuable insight into the potentially limited role of CSR, the robust impact of circadian timing, and the potential for circadian timing of therapy (chronotherapy), in influencing fasted physiology.

One of the aims of the current study was to determine whether CSR had any impact on subjective hunger ratings, as previous findings have shown sleep restricted individuals eat more calories than non-sleep restricted individuals (14–17). While we did not find a difference between groups in subjective hunger, we did find a robust circadian rhythm in hunger levels, which agrees with another study using validated circadian protocols (34). In that study, Scheer and colleagues found that levels of subjective hunger, independent of the wakefulness/sleep cycle, and thus the feeding/fasting cycle, follow a circadian rhythm such that individuals are most hungry in the evening hours and least hungry in the early morning hours (34). Carnell and colleagues have also shown a diurnal rhythm to hunger levels in obese individuals, with subjective hunger being higher in the evening as compared to the morning hours, coinciding with lower levels of peptide YY (satiety hormone) and higher levels of ghrelin (46). Our subjective hunger results display a similar peak (~1900 hours) and trough (~0700 hours), but we now add the additional findings that CSR does not impact this rhythm. The lack of a significant difference in the hunger rhythm during CSR is of note as it may add insight into why sleep restriction leads to excess caloric intake, particularly at night; sleep restricted individuals may have a higher likelihood of being awake across a longer duration of time when their internal physiology is promoting hunger if sleep is initiated at a later time. Subjective hunger does not begin to decline until between 1900 and 2300 hours, but still remains relatively high during this time. Thus, if one individual were to go to sleep at 2100 hours and another at 2300 hours, the drive for food consumption would still be reasonably high in that second individual during those two additional hours of wakefulness, and this may be driving the excess food consumption and weight gain observed during sleep restriction (14–17). It is unknown, however, if these hunger rhythms can be shifted by the light or other exogenous factors; this should be explored in future research.

In addition to not finding a difference between groups and a circadian rhythm in subjective hunger levels, we also did not find any significant differences between groups or significant rhythms in the fasted concentrations of circulating leptin and ghrelin. Leptin has been observed to follow a diurnal rhythm under fed conditions with concentrations lowest in the mid-afternoon, beginning to rise at ~2200 hours, and highest during the nighttime hours (~0300 hours) (35); however, these concentrations are greatly influenced by caloric intake (39) and sleep (40) and have not demonstrated circadian rhythmicity in a FD circadian protocol (47). Ghrelin has been reported to follow a diurnal rhythm with increases in the early portion of the night and decreases in the second half of the night with a trough in the morning at ~0800 hours (36–38), though these rhythms are blunted with sleep restriction suggesting that ghrelin is influenced by both the circadian and the sleep and wakefulness systems (36, 37). Our findings in leptin and ghrelin may differ from previous FD studies because we only measured fasted concentrations and there is a non-linear interaction between circadian and behavioral effects in leptin concentrations (47). Future work is needed to examine CSR in both the fed and fasted states to fully tease apart the influence of sleep restriction and circadian timing, particularly since levels of ghrelin in sleep restricted individuals have been found to predict subsequent caloric consumption (22).

Our findings of no difference in fasted insulin and glucose concentrations between the two conditions were somewhat surprising. Decreases in glucose tolerance and insulin sensitivity during acute insufficient and fragmented sleep protocols have been well established (48–52), and fasted insulin has been found to be lower than baseline levels after weeks of sleep restriction and circadian disruption (31). However, by using fasted levels and not oral or intravenous glucose tolerance tests as has been done previously in acute sleep restriction, we may not have challenged the system enough to see appreciable differences. Another hypothesis for why we did not observe differences between our two groups is that in previous sleep restriction studies, researchers may have been studying sleep restricted individuals at a different circadian phase than when the individuals were not sleep restricted (for example, if awakening were earlier in the sleep restriction condition), as circadian timing can independently play a role in impaired glucose metabolism (53, 54). Further supporting the premise that sleep restriction protocols may lead to studying individuals at a different circadian phase, in a previous protocol of 5 days of 5 h sleep restriction, impairments in early morning insulin sensitivity were found to be highly correlated with the circadian timing of high melatonin concentrations (55). In addition, protocols providing exogenous melatonin have found decreases in insulin secretion and sensitivity (56, 57), and individuals carrying a common variant in the melatonin receptor 1b gene have increased risk for impaired glucose regulation (58, 59), further supporting the hypothesis relating the circadian timing of melatonin to impaired glucose regulation. Our findings of no significant difference between conditions in adiponectin concentrations is in agreement with the literature that sleep restriction has limited impact on adiponectin (60, 61), though sleep restriction has been found to influence adiponectin differently depending on sex and ethnicity (61). Our limited sample size did not allow for in-depth examination of the impact of sex and ethnicity during CSR, and should be examined in future research. Finally, our findings of a non-significant trend for higher fasted cortisol in the CSR condition adds to the somewhat inconclusive literature on the impact of sleep restriction on cortisol, as acute sleep restriction has been reported to either elevate (49, 62, 63) or not change (64–66) cortisol concentrations. However, our data are in agreement with previous findings of no change in cortisol across weeks of sleep restriction and circadian disruption (31), but we now include a Control condition to add additional evidence to the limited impact of sleep restriction to the robust circadian rhythmicity of cortisol.

Our study did have several limitations and the conclusions should be considered with caution. First, we only measured the fasted hormone concentrations of the observed measures. It is unknown how these hormones would change after food consumption during CSR, which may be of importance as (i) individuals tend to ignore satiety signals during acute sleep restriction and continue to eat (15) and (ii) circadian timing without sleep restriction has been shown to dampen leptin levels when measured in the fasted and post-prandial state (47). Furthermore, we only had one fasted draw per wake episode. Additional draws may have added a temporal resolution to help detect more sensitive differences. Second, our population consisted of healthy young participants whose physiology may be able to respond quickly to the physiological challenge of sleep restriction. Though our protocol included a chronic exposure to insufficient sleep spanning across 32-days, it may not have been long enough or a sufficient amount of sleep restriction to see a metabolic inability to respond. Finally, our somewhat small sample size may have limited our ability to observe subtle differences between the two groups and as mentioned previously, reduced our capability to draw conclusions in regard to sex or ethnic differences in response to sleep restriction.

In summary, our findings demonstrate that sleep restriction may have a limited impact on hunger and fasted concentrations of hormones and that circadian timing needs to be considered when examining fasted levels of these outcomes. This information is important for clinicians who may need to make recommendations or prescribe therapies during the early morning hours and our findings could be used to help guide future chronotherapies.

## Data Availability Statement

The raw data supporting the conclusions of this manuscript will be made available by the authors, upon request, to any qualified researcher.

## Ethics Statement

This study was carried out in accordance with the recommendations of, and with the approval of, the Partners Healthcare Institutional Review Board (IRB #2011P001094). All subjects gave written informed consent in accordance with the Declaration of Helsinki.

## Author Contributions

EK obtained funding. EK and JH conducted design, data collection, data analysis, data interpretation, and writing. AM contributed to data collection, data analysis, data interpretation, and writing. CM contributed to data analysis, data interpretation, and writing.

## Conflict of Interest Statement

AM and CM have nothing to disclose; JH is currently employed by Vanda Pharmaceuticals Inc.; EK has received travel reimbursement from the Associated Professional Sleep Society and has served as consultant for Pfizer Inc. and in cases involving transportation safety and sleep deprivation. The reviewer SB and handling Editor declared their shared affiliation.
